# Spectrum of Febrile Thrombocytopenia in the Pediatric Population (1-18 Years) Admitted in a Tertiary Care Center

**DOI:** 10.7759/cureus.45681

**Published:** 2023-09-21

**Authors:** Trupti Deshpande, Harsh B Gupta, Nimisha Pandya, Shruti G Sethia, Lalit Nainiwal, Soumitra Sethia

**Affiliations:** 1 Pediatric Medicine, Nirmal Hospital Private Limited, Surat, IND; 2 Pediatric Medicine, Gujarat Medical Education and Research Society (GMERS) Medical College, Vadodara, IND; 3 Pediatrics and Neonatology, Gujarat Medical Education and Research Society (GMERS) Medical College, Vadodara, IND; 4 Community Medicine, Nandkumar Singh Chouhan Government Medical College, Khandwa, IND

**Keywords:** pediatric age, thrombocytopenia, febrile, dengue fever, bleeding manifestation

## Abstract

Aim

Thrombocytopenia is a common manifestation of various infections. Thrombocytopenia associated with fever helps to narrow down the differential diagnosis and management of fever. It also helps to know the various complications of thrombocytopenia, its management, and the outcome of the patient. This study aimed to evaluate the clinical profile and determine etiology and complications in patients with fever and thrombocytopenia in pediatric populations.

Methods

One hundred and fifteen patients of both sexes aged 1-18 years with fever and found to have thrombocytopenia (platelet count < 1.5 lakhs) between June 1, 2018 and March 31, 2019 were included in this study.

Results

Infection was the common cause of febrile thrombocytopenia and dengue fever was the most common infection. Bleeding manifestations were seen in 9.6 % of patients. Petechiae/purpura was the commonest bleeding manifestation followed by gum and nose bleeding. Common bleeding manifestations were seen in patients with a platelet count below 50,000 and the majority of them did not require platelet transfusion. Good recovery was noted in 96.5% of patients while 2.6% had mortality.

Conclusions

An infection, particularly dengue, was the common most cause of fever with thrombocytopenia. In the majority of patients, thrombocytopenia was transient and asymptomatic. Bleeding was present in the majority of patients with platelets less than 10,000 and 20,000 to 50,000. The most common bleeding manifestation was petechial rashes over the skin. Platelet transfusion was not required in most of the cases. On treating the specific cause, a drastic improvement in the platelet count was noted during discharge and further follow-up. Immunization is highly recommended for vaccine-preventable diseases.

## Introduction

Fever is the most common manifestation of an illness that occurs in children which makes parents seek early medical attention. Fever has been recognized as a cardinal manifestation of disease since ancient times, as recorded by ancient scholars like Hippocrates and Akkadain [1,2]. Fever is defined as an elevation of the body temperature above the normal circadian range as the result of a change in the thermoregulatory center located in the anterior hypothalamus and occurs in conjunction with an increase in the hypothalamic set point e.g. 37°c to 39°c. An A.M temperature of >37.2°C (98.9°F) or a P.M. temperature of >37.7°C (99.9°F) [3].

Most cases of prolonged fever are instances of well-known diseases manifesting them atypically. The actual pattern of graphic recording of fever is so variable that it is not helpful in pointing to specific diagnosis at all times; hence, an aggressive diagnostic effort is usually justified so that curative or palliative measures can be brought into use once the diagnosis has been achieved. Thrombocytopenia is defined as the platelet count < 1, 50,000/microliter. This is due to decreased production, increased destruction, and increased sequestration in the spleen [4]. Thrombocytopenia is characterized by bleeding most often from small vessels. This can manifest as petechiae over the skin and hemorrhages from the mucosa of gastrointestinal and genitourinary tracts. Rarely, the platelet count may be as low as 5,000/mm^3^ predisposing the patients to life-threatening bleeding in the central nervous system (CNS) or from the gastrointestinal and genitourinary tracts [5]. Platelets are tiny blood cells that help our body form clots to stop bleeding. Fever with thrombocytopenia narrows the differential diagnosis of the clinical entity. India is a tropical country and is home to a variety of infectious diseases due to climate favorable for the transmission of most of these infections. In recent years, with the onset of monsoon, a rising trend has been observed in the number of cases of febrile thrombocytopenia of varied etiology. The causes of thrombocytopenia are varied ranging from idiopathic, infectious to inflammatory. Infections, like malaria, dengue, leptospirosis, typhoid, viral fever, and septicemia, are few of the common causes of fever with thrombocytopenia [3]. Patients with fever with thrombocytopenia can initially present with just simple fever and may in due course of time lead to adverse unpredictable outcomes such as intra-cerebral bleeding, hemorrhage into vital organs, shock, and finally leading to death. This uncertain course is often a source of concern to the patients and treating doctors. Therefore, a well-organized systematic approach with an awareness of causes of fever with thrombocytopenia can shorten the number of investigations and bring out diagnosis preventing further fatal outcomes. In Gujarat, changes in climate such as temperature, humidity, rainfall, soil moisture, and the rising sea level are favorable for vector-borne disease transmission. Heavy rainfall which results in puddles provides good breeding conditions for vectors which leads to transmission of diseases like malaria, dengue fever, chikungunya, and lymphatic filariasis [6]. The study aimed at filling the gap of knowledge in this part of Gujarat regarding the cases of fever with thrombocytopenia. This study was planned with the objectives of evaluating the clinical profile and determining etiology and complications in patients with fever with thrombocytopenia. Patients presenting fever with thrombocytopenia may or may not have varied bleeding manifestations as per different platelet counts. 

## Materials and methods

This cross-sectional study was planned to achieve the objectives of the study. This study was conducted on children aged between 1 and 18 years from 1st June 2018 to 31st March 2019 at a tertiary care center in Gujarat state. The sample size was calculated by taking the prevalence of thrombocytopenia as 65% in fever cases, with a confidence interval of 95%, and taking the design effect as 1. The sample size was initially 106 with an additional 10% in the case of a possible loss to follow-up during the study; the revised sample size was 115. In the study area, every month around 10-15 new cases of fever with thrombocytopenia are detected. All Patients of both sexes aged 1-18 years with fever (temperature >99.90 F) and thrombocytopenia (platelets count < 1.5 lac) were included in the study. Patients with thrombocytopenia who were already diagnosed to have hematological disorder/malignancy, on treatment with chemotherapy and other immunosuppressants, diagnosed cases of platelet disorders and dysfunction, patients on treatment with antiplatelet drugs and other drugs causing thrombocytopenia, and patients with cirrhosis and chronic liver disease were excluded from the study. The study was carried out after explaining details regarding the study to guardians or parents by giving them PIS (Patient Information Sheet) and informed written consent was taken from guardians or parents. The interview was conducted using a questionnaire when the patient was admitted to the hospital, and details about past history, family history, and birth history, some details like chief complaints, general examination, systemic examination, laboratory investigations, and treatment profile were also registered. The final diagnosis was made after necessary investigations like complete blood count (CBC), dengue IgG, IgM, and NS1 by ELISA. Baseline platelet counts were done on day 0 (on the day of admission) and repeat platelet was done depending on the clinical condition of the patient (if bleeding tendency). Changes in the platelet count with clinical manifestations were recorded (>1.5, 1-1.5, <50 lakhs), and bleeding signs like epistaxis, hematuria, melena, etc. appeared. All the variables were grouped as per mathematic transformation of them into nominal /ordinal /interval &ratio. Further point estimates with dispersion measures were calculated with the help of MS Excel & other statistical software. The extent of type one error was measured with parametric analysis. The Z test/Chi Sq test was applied for proportion and the t-test will be used to find out any significant difference between among detected proportion & mean. The 2012 WHO criteria were used to classify dengue according to levels of severity: 1. Dengue without warning signs; 2. Dengue with warning signs (abdominal pain, persistent vomiting, fluid accumulation, mucosal bleeding, lethargy, liver enlargement, increasing hematocrit with decreasing platelets); and 3. Severe dengue (dengue with severe plasma leakage, severe bleeding, or organ failure). Patients who recover following defervescence are considered to have non-severe dengue, but those who deteriorate tend to manifest warning signs. These individuals are likely to recover with intravenous rehydration. However, further deterioration is classified as severe dengue, though recovery is possible if appropriate and timely treatment is given.

## Results

Fever with thrombocytopenia is one of the most challenging problems in children in the field of medicine. Fever with thrombocytopenia consists of occult presentations of common diseases rather than rare diseases. A total of 115 patients with febrile thrombocytopenia were included in this study during the period from June 1, 2018, to March 31, 2019 (no missed out cases). The age range of the patient was between 1 year and 18 years. The majority of the patients (65%) were below 10 years of age. The majority of cases (more than 75%) were seen in the post-monsoon period. The majority of study participants (79%) were males as compared to females (21%). The average stay of patients in the hospital was 3.63 with SD 2.57 days. Ninety percent of patients stayed less than five days for their illness. The distribution of the study population according to the clinical features of diseases is depicted in Table 1.

**Table 1:** Distribution of the study population according to clinical features of diseases

Sr. No.	Clinical features	Frequency	Percentage
1	Fever	115	100
2	Fever with chills and rigors	27	23.5
3	Generalized weakness and body ache	70	60.9
4	Abdominal distention	55	47.8
5	Vomiting	42	36.5
6	Abdominal pain	30	26.1
7	Cough and common cold	18	15.6
8	Headache	12	10.4
9	Petechiae on the skin	9	7.8
10	Poor oral intake	8	6.9
11	Loose stool	4	3.6
12	Convulsion	3	2.6
13	Bleeding from nose	2	1.7

In this study, patients had different signs like ascites (40.9%), generalized edema (39.1%), decreased urine output (39.1%), icterus (17.4%), petechial rashes (7.8%), and red flush (5.2%). In general examination at the time of admission, 35.6% of patients had raised temperature (fever), 1.7% of patients had hypothermia, 32.2% of patients had abnormality in pulse (low volume pulse in 13.5% and palpable bounding pulse in 86.5% patients), 18.3% of patients had hypotension, and 3.5% of patients were on ventilation due to their critical illness. In systemic examination, maximum abnormal findings were detected in the gastrointestinal tract (57.3%) whereas in the CNS, it was 8.7%. Out of total abnormality gastrointestinal system (GI), the most common abnormalities were abdominal distention (83.3%), ascites (71.2%), and hepatomegaly (40.9%) followed by abdominal tenderness (20%) and splenomegaly (15%). Out of total abnormality in the CNS, 70% had altered sensorium, and 30% had restlessness. Out of all study participants, the majority of patients (78.3%) had dengue fever, of which 46.1% patients had dengue without warning signs, 28.7% patients had dengue with warning signs, whereas 3.5% patients had severe dengue. Out of total dengue cases, 70%, 16.7%, and 13.3% cases were diagnosed by Dengue NS1Ag, Dengue IgM, and Dengue IgG serological tests, respectively. The distribution of the study population according to different types of etiology is depicted in Table 2.

**Table 2:** Distribution of the study population according to different types of etiology

Sr. No.	Final Diagnosis	Types	Number	Percentage
1	Dengue fever (90)	Without Warning signs	53	46.1%
		With Warning signs	33	28.7%
		Severe Dengue	4	3.5%
2	Septicemia (7)	Without Shock	2	1.7%
		With Shock	5	4.3%
3	Malaria (8)	Plasmodium Vivax	4	3.5%
		PlasmodiumFalciparum	4	3.5%
4	Enteric fever		4	3.5%
5	Acute Lymphoid Leukemia (ALL)		3	2.6%
6	Hemophagocytic Lymphohistiocytosis (HLH)		2	1.7%
7	Lupus nephritis		1	0.9%
Total			115	100%

Among all study populations (115), 37% of patients had anemia out of which 15.7% had moderate anemia, 13.9% had mild anemia, whereas 7.8% patients had severe anemia.

The distribution of patients according to etiology and different platelet counts is depicted in Table 3.

**Table 3:** Distribution of patients according to etiology and different platelet counts ALL: Acute lymphoid leukemia; HLH: hemophagocytic lymphohistiocytosis

Sr. No.	Etiology	Less than 10,000	10,000 to 20,000	20,000 to 50,000	50,000 to 1,00,000	More than 1,00,000	Total
1	Dengue	6.7%	6.7%	24.4%	25.6%	36.7%	100% (90)
2	Malaria	12.5%	12.5%	37.5%	25.0%	12.5%	100% (8)
3	Septicemia	14.3%	0.0%	28.6%	42.9%	14.3%	100% (7)
4	Enteric fever	0.0%	0.0%	25.0%	0.0%	75.0%	100% (4)
5	ALL	0.0%	66.7%	33.3%	0.0%	0.0%	100% (3)
6	HLH	0.0%	0.0%	50.0%	50.0%	0.0%	100% (2)
7	Lupus Nepritis	0.0%	0.0%	0.0%	0.0%	100.0%	100% (1)
Total		7.0% (8)	7.8% (9)	26.1% (30)	25.2% (29)	33.9% (39)	100%(115)

Out of a total of 115 patients, 9.6% of patients had different types of bleeding manifestations. The most common bleeding manifestation was petechial rashes over the skin (81.8%), whereas 18.1% each had bleeding from the nose and gum, and 9.1% each had bleeding from the ear and hematuria. One patient had more than one type of bleeding manifestation. There was a significant association between the platelet count being less than 50,000 and bleeding tendency with a p-value of 0.024 (<0.05) and a chi-square value of 5.10. Bleeding was present in the majority of patients with platelets less than 10,000 (27.3%) and 20,000 to 50,000 (27.3%). In this study, 67.3% of patients with bleeding manifestations had dengue followed by 27.3% who had malaria and 6.1% who had septicemia. Other rare etiologies (like enteric fever, HLH) of fever with thrombocytopenia had no bleeding manifestations. 96.5% of patients were discharged from the hospital with good recovery (mean 3.5 days for improvement in the platelet count), whereas 2.6% of patients died of septicemia with shock and 0.9% of the critically serious patient was discharged against medical advice (DAMA).

## Discussion

In this study, there was a total of 115 patients presenting with fever with thrombocytopenia in which the most common cause of febrile thrombocytopenia was dengue fever (78.3%), followed by septicemia (6.1%), malaria (6.9%), enteric fever (3.5%), and acute lymphoid leukemia (2.6%). The maximum number of cases (more than 75%) were seen in October (33%), November (19.1%), September (12.2%), and August (11.3%) i.e. post-monsoon season. Chakravarti et al. and Mistry et al. in their studies found that maximum cases of febrile thrombocytopenia had dengue fever and were seen in the post-monsoon period i.e. October. This is attributed to rainfall, ambient temperature, and humidity which causes an increased development rate of mosquitoes [7,8]. In this study, 65% of study participants were below 10 years of age, and the mean age of the study population was 8.2 with an SD of 4.75 years. Similar findings were observed in the study by Gutthi et al. in which 70% of patients were below 10 years [9], younger the age the more affected due to outdoor activities.

In this study, 79% of study participants were males as compared to 21% females. In studies conducted by Ramabhatta et al., Masamatti et al., and Gutthi et al., similar results were found wherein more males were affected than females possibly due to exposure to mosquitoes and outdoor activities [9-11]. In this study, we found that around 90% of patients with fever with thrombocytopenia had a hospital stay of less than five days with an average stay of three days. Out of the total patients, 4.3% patients had a stay of more than 10 days due to critical condition. Mishra et al. in their study regarding dengue fever found that the mean tenure of hospitalization was 3.8 days [12].

In this study, as per inclusion criteria, all the patients had fever. Common clinical features were generalized weakness and body ache (60.9%), abdominal distention (47.8%), vomiting (36.5%), abdominal pain (26.1%), fever with chills and rigor (23.5%), cough, common cold (15.6%), and headache (10.4%), whereas less common clinical features were petechia on the skin (7.8%), poor oral intake (6.9%), loose stools (3.6%), convulsion (2.6%), and bleeding from nose (1.7%). In the study by Gondhali et al., the most common symptoms were headache (90%), generalized weakness and body ache (92%), vomiting (43%), abdominal pain (24%), and sore throat (8%) [3]. Rao et al. found myalgia (64.7 %) and headache as the most common symptoms [13]. The study by Kumar et al. found vomiting (18.94%) as the commonest symptom followed by abdominal pain (16.31%) and loose motions (4.73%) cases [5]. Comparing this study and other studies, we found that GI symptoms were most commonly associated with febrile thrombocytopenia. This could be because of liver involvement and fluid accumulation in the peritoneal cavity in patients with dengue fever which is a common disease.

In this study, signs in general examinations at the time of admission were hyperthermia (35.6%) and hypothermia (1.7%). Hypotension was observed in 18.3% of patients with dengue with warning signs, severe jaundice, and septicemia with shock. In this study, the majority of patients (80%) had high-grade fever. Other common signs were ascites (40.9%), generalized edema (39.1%), decreased urine output (39.1%), icterus (17.4%), petechial rashes (7.8%), and red flush (5.2%). In systemic examination, patients had abnormality detected in the GI system (57.3%), CNS system (8.7%), respiratory system (1.7%), and cardiovascular system (CVS) (0.9%). Out of the total study population, common GI findings were abdominal distention (47.8%) and ascites (40.9%). Common CNS abnormalities were altered sensorium (6.1%). Gutthi et al. found that anemia was the most common sign in 69% of patients followed by hepatomegaly (41%) splenomegaly (32%), and lymphadenopathy (5%) [9]. The study by Gondhali et al. observed that 28% of patients had icterus, (22%) pallor, (19%) splenomegaly, (15%) altered sensorium, and 12% patients had hepatomegaly [3].

Dengue fever is classified into three categories. Out of the total study population (115), 78.3% of patients had dengue fever of which 46.1% of patients had dengue without warning signs, 28.7% of patients had dengue with warning signs, whereas 3.5% of patients had severe dengue. Among all the study populations, 6.1% of patients had septicemia. Patne et al., Gondhali et al., Modi et al., and Raikar et al. had similar findings to this study that viral infection like dengue fever is the most common cause of febrile thrombocytopenia [3,14-16], whereas Gandhi et al. and Kumbhar et al. found malaria as the common etiology for fever with thrombocytopenia [17-19]. Other rare causes include septicemia, enteric fever, and hematological malignancy like ALL. The pathogenesis of thrombocytopenia in dengue fever is increased peripheral destruction of antibody-coated platelets and bone marrow suppression. The incidence and prevalence of various infections vary seasonally and geographically. Some infectious diseases occur cyclically. The duration and period of a study conducted also affect the study results. Thrombocytopenia means a platelet count less than 1, 50,000 per microliter. In this study, all patients had a platelet count of less than 1,50,000 per microliter as per the inclusion criteria of the study. In this study, there was a significant association between the platelet count of less than 50,000 per microliter and bleeding tendency with a p-value of 0.024 (<0.05) and a chi-square value of 5.10. In this study, 40% of patients had a platelet count less than 50,000 (26.1%) between 20,000 and 50,000 per microliter, (7.8%) 10,000 to 20,000 whereas 7% had less than 10,000 per microliter. Bleeding was present in the majority of patients with platelet less than 10,000. Out of a total of 115 patients, the most common bleeding manifestation was petechial rashes over the skin (81.8%), and 18.1 % had nose and gum bleeds. In this study, the majority of patients (67.3%) with bleeding manifestations had dengue fever followed by malaria and septicemia. Gutthi et al. in their study found that out of 34 cases of dengue, mild thrombocytopenia was seen in 18%, moderate in 38%, and severe in 44% of cases [9]. Bleeding manifestations were seen in 45% of patients of which GI bleeding was common. Nair et al. and Modi et al. in their studies observed that major bleeding manifestations were having platelet <20000 [15,16] and the major bleeding manifestation was skin and mucosal bleeds. Regarding the outcome of illness, in this study, 96.5% were discharged from the hospital with good recovery whereas 2.6% of patients died, and 0.9% critically serious patient was DAMA. Similar studies done by Kumbhar et al. and Patil et al. showed a good recovery rate [19,20].

The study findings help us understand the fact that fever with thrombocytopenia may present in atypical ways, and immunological tests are needed for correct diagnosis and blood transfusion may not be needed even if the platelet counts are around 20,000.

This study has a few limitations. This study is applicable only to the pediatric age group and hence, we do not know whether the same results can be extrapolated to the general population in all age groups as well. The other limitation was many acute febrile cases may have been treated in the peripheral clinics and hospitals without any complete blood count being done at all. Hence, our hospital-based model might not reflect all the cases of fever with thrombocytopenia in the given locality or population. Other than the above limitations, we could not assess the information bias, inability to assess temporal relationships, or lack of confounders though we tried to minimize them.

## Conclusions

Fever with thrombocytopenia is one of the most challenging problems with the occult presentation of common diseases rather than rare diseases in children. Infections like dengue fever, malaria, septicemia, and enteric fever are common causes of febrile thrombocytopenia. Dengue fever, malaria, and septicemia still present clinically in atypical and occult forms, making diagnosis more difficult, so a high index of other clinical suspicion is needed. Other than routine investigations, one should do some specific tests like card tests for malarial parasites, IgM for ELISA, NS1Ag for Dengue, Widal test, Typhidot IgM, blood culture, etc. are required for correct diagnosis. Immunization is recommended for vaccine-preventable diseases and preventive measures for vector-borne diseases.
